# Rapid CRISPR/Cas9-Mediated Cloning of Full-Length Epstein-Barr Virus Genomes from Latently Infected Cells

**DOI:** 10.3390/v10040171

**Published:** 2018-04-03

**Authors:** Misako Yajima, Kazufumi Ikuta, Teru Kanda

**Affiliations:** Division of Microbiology, Faculty of Medicine, Tohoku Medical and Pharmaceutical University, 1-15-1 Fukumuro, Miyagino-ku, Sendai 983-8536, Japan; yajima@tohoku-mpu.ac.jp (M.Y.); ikutak@tohoku-mpu.ac.jp (K.I.)

**Keywords:** Epstein-Barr virus (EBV), latent infection, CRISPR/Cas9, bacterial artificial chromosome (BAC), PacBio sequencing

## Abstract

Herpesviruses have relatively large DNA genomes of more than 150 kb that are difficult to clone and sequence. Bacterial artificial chromosome (BAC) cloning of herpesvirus genomes is a powerful technique that greatly facilitates whole viral genome sequencing as well as functional characterization of reconstituted viruses. We describe recently invented technologies for rapid BAC cloning of herpesvirus genomes using CRISPR/Cas9-mediated homology-directed repair. We focus on recent BAC cloning techniques of Epstein-Barr virus (EBV) genomes and discuss the possible advantages of a CRISPR/Cas9-mediated strategy comparatively with precedent EBV-BAC cloning strategies. We also describe the design decisions of this technology as well as possible pitfalls and points to be improved in the future. The obtained EBV-BAC clones are subjected to long-read sequencing analysis to determine complete EBV genome sequence including repetitive regions. Rapid cloning and sequence determination of various EBV strains will greatly contribute to the understanding of their global geographical distribution. This technology can also be used to clone disease-associated EBV strains and test the hypothesis that they have special features that distinguish them from strains that infect asymptomatically.

## 1. Introduction

Epstein-Barr virus (EBV) is a human lymphocryptovirus that establishes a life-long latent infection in the majority of healthy adults. EBV infection is mostly asymptomatic, but it sometimes causes human diseases, such as infectious mononucleosis, various lymphomas (B cell lymphomas and NK/T cell lymphomas), and epithelial cancers (nasopharyngeal carcinomas and gastric cancers).

EBV has a double-stranded DNA genome of approximately 175 kb, which is, like other herpesvirus genomes, relatively large. Thus, it is cumbersome to determine the entire EBV genome sequence using a conventional Sanger sequencing method. The very first EBV whole genome sequencing was totally dependent on several cosmid clones obtained from purified EBV virion DNAs [1].

The recent development of deep sequencing technology has dramatically changed the strategy of whole EBV genome sequencing [2]. It is now possible to use total genomic DNA from EBV-infected cells to extract EBV genome sequences. Various EBV strains derived from more than 70 EBV-positive cell lines were sequenced using a hybrid capture approach followed by sequencing with short-read sequencers (Illumina HiSeq 2000) [3]. However, short-read sequencers cannot determine repetitive sequences in the EBV genome. Such repetitive sequences include internal repeats 1 through 4 (IR1–4), family of repeats (FR), and several coding sequences of EBV nuclear antigens (EBNAs) [4]. As a result, the obtained sequences have a number of gaps. Since some of the repetitive regions affect EBV viral functions, an alternative experimental strategy to determine the complete sequences of various EBV strains is highly desirable. 

Another way to extract EBV DNA from latently infected cells is to clone the EBV genome in a bacterial artificial chromosome (BAC) vector, a cloning vector that can accommodate more than 200 kb of DNA. BAC cloning of EBV genomes enables the preparation of unlimited amounts of viral DNA that can readily be subjected to deep sequencing. EBV-BAC cloning was pioneered by Professor Wolfgang Hammerschmidt and colleagues using B95-8 strain EBV [5]. The group used a conventional gene targeting strategy to insert a BAC vector sequence into the EBV episomes. This study was followed by a number of studies that utilized similar approaches to clone several EBV strains [6,7,8]. A conventional EBV-BAC cloning strategy was recently modified and applied to more EBV strains, including some disease-associated strains [9,10]. 

Clustered regularly interspaced short palindromic repeats (CRISPR)/Cas9 system are derived from an RNA-guided sequence-specific prokaryotic antiviral immune system, and the system has now been applied to develop various antiviral strategies [11,12,13,14]. In the case of viruses that integrate into human chromosomes (for example, proviruses of human immunodeficiency virus (HIV)), the viruses were successfully clipped out using the CRISPR/Cas9 system [15]. CRISPR/Cas9 system is also effective for eliminating viral episomes from latently cells. For example, targeting critical EBV genes, such as *oriP*, *EBNA*, and *LMP1* genes, resulted in the elimination of EBV episomes from the infected cells [16,17].

CRISPR/Cas9-mediated genome editing has the potential to revolutionize DNA engineering of herpesvirus genomes in latently infected cells. The technique was adapted to introduce a specific deletion into the regulatory region of EBV microRNAs [18]. Efficient gene knock-in techniques have also been developed for herpesviruses. Nonhomologous end joining was used to introduce a transgene into the Pseudorabies virus genome [19] and avian herpesvirus genome [20], while homologous recombination was used to introduce a small transgene into the EBV genome [18]. Homologous recombination, rather than nonhomologous end joining, is ideal for inserting transgenes to specific loci without compromising viral functions.

We came up with the idea of utilizing genome editing technology for EBV-BAC cloning. For EBV-BAC cloning, transgenes of more than 12 kb should be inserted into the EBV genome, which is a technical challenge. We recently invented a technology to rapidly clone EBV genomes using CRISPR/Cas9-mediated homology-directed repair [21]. Others also utilized CRISPR/Cas9 to rapidly clone the genome of the Pseudorabies virus [22]. While others use linear DNAs as donors [22], we use circular donor plasmids [21], which makes our strategy very straightforward. In this review, we focus on our BAC cloning strategy of EBV genomes, whereby CRISPR/Cas9 plasmid and circular donor plasmids are simultaneously transfected. This experimental strategy has greatly facilitated EBV genome cloning and whole viral genome sequencing including the sequencing of repetitive regions. We discuss how this technique may contribute to future genetic studies of EBV.

## 2. Concept of CRISPR/Cas9-Mediated EBV Genome Cloning

EBV genomes are maintained as circular, double-stranded DNA molecules (episomes) of approximately 175 kb in latently infected cells. To clone the EBV genome into a BAC vector, a BAC vector sequence must be inserted into the EBV genome.

The CRISPR/Cas9-mediated EBV cloning arose initially from the idea that cutting the EBV episome at a single site would stimulate homology-directed repair and increase the efficiency of transgene insertion. It was previously reported that CRISPR/Cas9-mediated knock-in of a large DNA sequence can be done by using a donor plasmid with relatively short homology arms (approximately 500-bp) [23]. Thus, it is expected that the homology arms could be made much shorter than with conventional methods.

**Figure 1:**
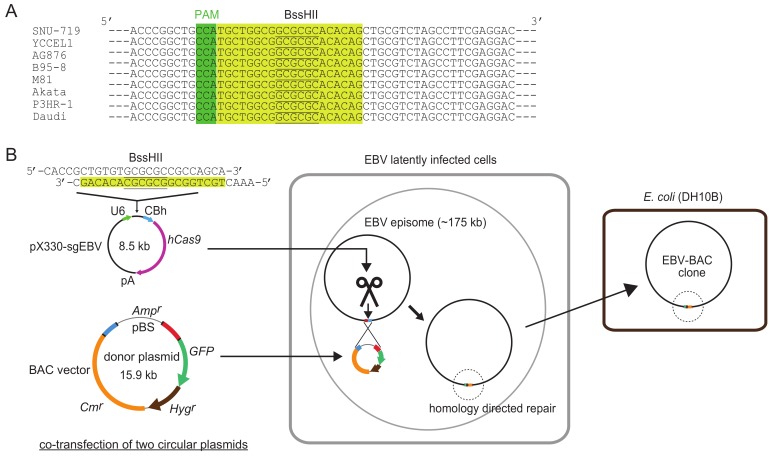
Schematic illustration of clustered regularly interspaced short palindromic repeats (CRISPR)/Cas9-mediated Epstein-Barr virus (EBV)-bacterial artificial chromosome (BAC) cloning. (**A**) CRISPR/Cas9 target sequence used for EBV-BAC cloning. Twenty bp sequences are shaded in yellow, and PAM sequences (5′-NGG) are shaded in green. BssHII sites are underlined. Note that the target sequence is 100% conserved among representative EBV strains so far sequenced. (**B**) Outline of EBV-BAC cloning. EBV latently infected cells are transfected with two circular plasmids (pX330-sgEBV and a donor plasmid). CRISPR/Cas9-mediated cleavage of EBV genome DNA stimulates homology-directed repair, and transgenes are integrated into EBV episomes via homologous recombination. EBV-BAC clones can readily be obtained by transforming DH10B *E. coli* with episomal DNA.

Figure 1 illustrates how the system works. A highly conserved 20-bp sequence, followed by a PAM (protospacer adjacent motif) sequence (5′-NGG), within the transgene insertion locus is used as a CRISPR/Cas9 target sequence. Two circular plasmids, a CRISPR/Cas9 plasmid and a donor plasmid, are transfected into EBV latently infected cells, and a BAC vector sequence and marker genes are inserted into the EBV genome via homology-directed repair. Once a BAC vector sequence is inserted into the EBV genome, EBV-BAC clones can readily be obtained by using episomal DNA for *E. coli* transformation.

## 3. Choosing the Transgene Insertion Site

**Figure 2:**
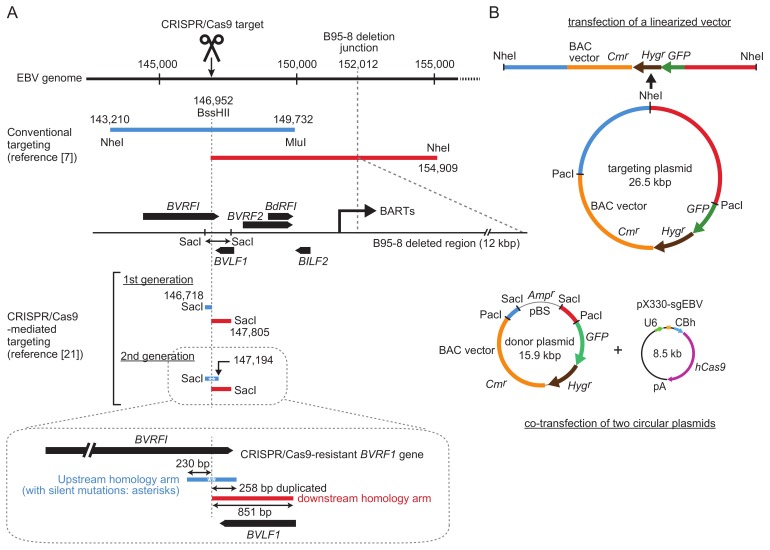
Comparison of homology arm lengths used for conventional and CRISPR/Cas9-mediated EBV-BAC cloning. (**A**) Homology arms employed by conventional targeting [24] and CRISPR/Cas9-mediated targeting [21] are illustrated by preserving their relative sizes. Upstream homology arms are in blue, and downstream homology arms are in red. Nucleotide numbers correspond to those of the B95-8 strain of EBV (V01555.2). EBV ORFs around the transgene insertion site are indicated as black arrows. Restriction enzyme sites used for targeting vector construction are indicated. Upstream and downstream homology arms of the second generation donor plasmid, together with the locations of *BVRF1*/*BVLF1* ORFs, are illustrated in detail at the bottom. (**B**) Schematic representation of a targeting plasmid (top, for conventional targeting) or a donor plasmid (bottom, for CRISPR/Cas9-mediated targeting). Note their different sizes.

EBV genomes are packed with more than 80 viral open reading frames (ORFs) [4]. B-cell transforming ability as well as progeny virus production ability needs to be preserved after transgene insertion. From this viewpoint, there are only a few transgene insertion sites that have been verified to have a minimal effect on viral functions. The insertion site used in conventional EBV genome targeting is near the junction of the so-called B95-8 deleted region [5,7] (Figure 2). This locus was selected as a transgene insertion site since the B95-8 strain of EBV, a common laboratory EBV strain, has a 12 kb deletion at this locus that does not compromise viral functions. The locus is upstream of the promoter region of BamHI A rightward transcripts (BARTs), and transgene insertion into this region preserves BART microRNA expression [24].

The *BXLF1* (viral thymidine kinase) locus was also used as a transgene insertion site. Recombinant EBVs of Akata strain, with a neomycin resistance gene inserted to the locus, works nicely in infection experiments [25,26]. When the Akata strain EBV genome was cloned into a BAC vector, the same *BXLF1* locus was chosen as an insertion site of a BAC vector sequence and a neomycin resistance gene [8]. However, the neomycin resistance gene in the BAC clone DNA worked poorly in transfected cells for unknown reasons [8]. This observation highlights that a BAC vector insertion site should be carefully chosen.

Recently, the terminal repeat (TR) region of the EBV genome was used as an insertion site. EBV M81 strain, a virus isolated from a Chinese patient with nasopharyngeal carcinoma, was first used [10], followed by several EBV-positive cancer cell lines [9]. A BAC vector sequence and a hygromycin resistance gene were recombined into the TRs of EBV strain M81. A linear targeting vector, having four copies of TR on both ends, was used for the targeting [9]. While transgene insertion into the terminal repeat region does not disrupt any viral coding sequences, it disrupts the expression of latent membrane protein (LMP) 2, as exons of the *LMP2* gene are separately located at both sides of the TRs. Importantly, transgenes inserted to the TR region are auto-excisable [10,27]. When lytic replication cycle is induced, TRs are subject to random cleavage, followed by homologous recombination-mediated joining [10,27]. During this process, the inserted transgenes are stochastically removed from the EBV genome. It was found that B cells infected with recombinant M81 virus exclusively carried viral episomes that were devoid of the transgenes [10], implicating that viral episomes with LMP2A expression were selected in immortalized B cells. Since LMP2A is an important latent viral protein in the pathogenesis of the virus [4], autoexcision of the transgenes is advantageous. However, a drawback of the system is that the hygromycin resistance gene can no longer be used for further infection experiments.

Figure 2 shows a comparison of homology arms (upstream and downstream) used for either conventional targeting or CRISPR/Cas9-mediated targeting. In conventional targeting, the homology arms are approximately 5–10 kb [5,7,24]. Long homology arms are recommended to maximize homologous recombination efficiency. In addition, a transgene cassette, consisting of a BAC vector sequence and marker genes (a drug resistance gene and a GFP gene), is included between these long homology arms, making the targeting vectors quite large (up to 26.5 kb, as shown in Figure 2) [24].

For CRISPR/Cas9-mediated targeting, according to our previous experience of conventional targeting of the EBV B95-8 strain, the same *BVRF1*/*BVLF1* region was chosen as a transgene insertion site. This region contains a 1088-bp SacI fragment that provides the upstream and downstream homology arms for targeting. The CRISPR/Cas9 target sequence (Figure 1) is within the SacI fragment, and it overlaps with the BssHII site. The upstream and downstream homology arms are 230 and 851 bp, respectively, which are much smaller than those used for conventional targeting (Figure 2). CRISPR/Cas9-mediated cleavage apparently disrupts the *BVRF1* ORF, but the ORF can be reconstituted by designing a specific donor plasmid (see below).

Other transgene insertion sites may also work. The EBV genome annotation has been curated by Professor Paul Farrell. He has created a graphical map (an excel file) of EBV-wt (NC_007605.1) [28], and the file can be downloaded from his web site (www.imperial.ac.uk/people/p.farrell). The graphical map shows all the known EBV ORFs (with transcription start and stop sites indicated) and the long range splicing as well. The ideal insertion site should be free of EBV ORFs, regulatory regions, and long range splicing. From this point of view, the region between *BBRF3* and *BBLF1* could be a candidate site for the transgene insertion. It is now becoming clearer that noncoding RNAs are encoded by EBV genome regions that were considered to be free of viral genes [29,30,31]. Thus, one should always keep it in mind that transgene insertion into the EBV genome can disrupt such noncoding RNA expression. 

## 4. How to Design Donor Plasmids Harboring Transgenes

In conventional targeting experiments, targeting vectors are linearized prior to transfection into EBV-positive cell lines (Figure 2). Thus, one must design a targeting vector that can be linearized by a restriction enzyme (for example, NheI in Figure 2). This limitation complicates the design of the targeting vector. Linear targeting vector may also be obtained by long-range high fidelity PCR, but it may not be completely accurate. Since upstream and downstream homology arms are quite long, the sequences of the homology arms vary between EBV strains. Therefore, to use targeting vectors with 100%-matched homology arms, a targeting plasmid must be made for every single EBV strain, which is laborious. This is probably the major reason why only a few EBV strains have been cloned using conventional targeting.

In CRISPR/Cas9-mediated targeting, donor plasmid construction is simple due to the short homology arms. A detailed experimental strategy for constructing a donor plasmid was described in our original publication [21]. Briefly, the 1088-bp SacI fragment is first cloned into a cloning vector (pBluescript), and then a transgene cassette (a PacI fragment containing a BAC vector sequence, a *GFP* gene, and a hygromycin resistance gene) is cloned into the PacI site, created at the position of the BssHII site (Figure 2). The upstream homology arm is very well conserved among various EBV strains, while a little heterogeneity is observed in the downstream homology arm. This is also the case for two EBV-positive gastric cancer cell lines (SNU-719 and YCCEL1), which we used as the first test cell lines. Thus, two donor plasmids were prepared. Importantly, since the homology arms are quite short, these donor plasmids can be reused to clone many other EBV strains as well (see below). 

A critical decision was whether to use a circular donor plasmid or a linearized one. Although linearized DNA fragments are commonly used for conventional gene targeting experiments, circular donor plasmids are recommended for gene knock-in experiments in mice [32]. It is well known that higher transfection efficiency can be obtained by using circular plasmids. The donor plasmid was designed so that we could test both linear and circular states of the plasmid. The circular donor plasmid can be used as it is, or it can be digested by SacI to obtain a linear fragment spanning the homology arms and the transgenes (Figure 2). 

Our initial attempt was to introduce a single cut to the target site and simply insert transgenes into the cutting site. The EBV genome cleavage site is located within the *BVRF1* gene, which encodes an essential minor capsid protein. When the first-generation donor plasmid was used for transgene knock-in, the *BVRF1* gene was disrupted [21]. Thus, we constructed a second-generation donor plasmid. In this donor plasmid, the upstream homology arm was extended to include the *BVRF1* stop codon and polyadenylation signal (Figure 2, illustrated in detail at the bottom). To make the extended upstream homology arm resistant to CRISPR/Cas9, we introduced silent mutations into the CRISPR/Cas9 target sequence within the *BVRF1* ORF [21]. The mutated gene encodes exactly the same *BVRF1* amino acids as the wild-type gene. The upstream and downstream homology arms of the second-generation donor plasmid have 238-bp sequence duplication. We found that the transgene knock-in was not disturbed by this sequence duplication [21]. Homologous recombinants with intact *BVRF1* expression were successfully obtained by using the second-generation donor plasmid. 

## 5. Preparing Donor Plasmids to Clone Various EBV Strains

**Figure 3:**
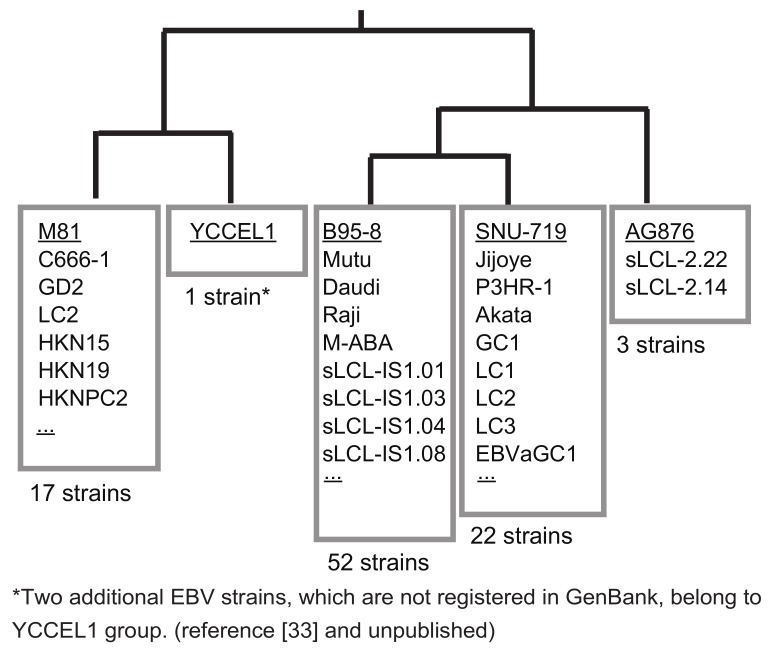
EBV strains can be categorized into five groups based on homology arm sequences. The sequences of 1088-bp SacI fragments of representative EBV strains (AG876, SNU-719, B95-8, YCCEL1, and M81) were subjected to phylogenetic tree analysis (CLUSTALW) and BLAST searches to find out how this region is conserved among various EBV strains. The numbers of EBV strains whose SacI fragment was 100% identical to that of the representative EBV strains are indicated. Only the EBV strains with correctly annotated *BVRF1* and *BVLF1* genes are taken into account.

If one wants to clone a new EBV strain from a cell line, preparing a donor plasmid to be used for the specific EBV strain is very straightforward. In the case of a new EBV-positive cell line, the SacI fragment (1088-bp) (Figure 2) should first be PCR-amplified and sequenced either directly or after cloning the fragment in pBluescript. The donor plasmids for cloning either SNU-719 EBV or YCCEL1 EBV are available [21]. Thus, if the sequence is identical to either SNU-719 or YCCEL1, one can use the same donor plasmid. If not, the homology arms should be replaced to obtain a new donor plasmid. Nucleotide sequences of the 1088-bp SacI fragment of representative EBV strains were subjected to a BLAST search. The majority of EBV strains can be grouped into only five groups (Figure 3). Two Japanese-derived EBV strains, an EBV-positive natural killer cell line SNK6 [33] and a spontaneously established LCL, belong to the group of YCCEL1, although they are not deposited to GenBank (Figure 3). Thus, preparing just five donor plasmids allows cloning of the majority of EBV strains worldwide.

In summary, since donor plasmids with fairly short (200–800 bp) homology arms are used to get homologous recombinants, just a few donor plasmids are sufficient to clone hundreds of EBV strains worldwide.

## 6. How to Monitor the Cutting Efficiency of EBV DNA in Latently Infected Cells

pX330 [34], a commonly used CRISPR/Cas9 plasmid, was used to make pX330-sgEBV, which introduces a single cut to the target site (Figure 1). The target sequence of the CRISPR/Cas9 plasmid was chosen by subjecting the 1088-bp sequence to CRISPRdirect (http://crispr.dbcls.jp/). So far, the CRISPR/Cas9 plasmid did not show any off-target effects, which can be manifested by cell growth retardation or cell death of the host cells. Importantly, the target sequence of pX330-sgEBV is 100% conserved among all the EBV strains so far sequenced (Figure 1). Thus, this plasmid can be used to introduce a single cut in the genome of many EBV strains.

We used a GFP reporter (pCAG-EG-*ebv*-FP) to monitor cutting efficiencies of EBV DNA [32]. In pCAG-EG-*ebv*-FP, the target EBV DNA (1088-bp SacI fragment) was cloned between overlapping *EGFP* open reading frames. This reporter system turned out to be extremely useful to evaluate the cutting efficiency of EBV DNA by CRISPR/Cas9 in various cell lines. The cutting efficiency was very high in HEK293 cells, moderately high in SNU-719 cells, and relatively low in YCCEL1 cells [21]. The system can also be used to check the cutting efficiency in other EBV-infected cells, such as EBV-transformed lymphoblastoid cell lines (LCLs) and NK/T lymphoma cells lines. Thus, the efficiency of obtaining homologous recombinants can roughly be estimated before cloning begins.

## 7. Detection of Homologous Recombinants

To clone EBV genomes in latently infected cells, two circular plasmids, pX330-sgEBV and a donor plasmid, should be transfected into EBV-positive cell lines. Since the donor plasmid contains a *GFP* transgene, GFP expression can be observed shortly after transfection. A circular donor plasmid exhibits a higher frequency of GFP-positive cells than a linear donor plasmid, supporting the idea that higher transfection efficiency can be obtained by using circular donor plasmids. The presence of homologous recombinants in genomic DNAs can be detected at three days post-transfection using PCR analyses [21].

Enrichment of GFP-positive cells, which is a good indicator of successful homologous recombination, can be observed within approximately three weeks after starting hygromycin selection. Isolation of GFP-positive cell clones, which is required for the conventional targeting, is not necessary. Episomal DNAs prepared from pools of GFP-positive cells give far more intense PCR bands representing homologous recombinants than those prepared from the cells at three days post-transfection. Importantly, enrichment of GFP-positive cells can be observed only when circular donor plasmids and pX330-sgEBV are co-transfected. GFP-positive cell clusters hardly appear in the absence of pX330-sgEBV transfection, indicating that CRISPR/Cas9-mediated cleavage of EBV DNA is indispensable for efficient homologous recombination. Another important observation is that only circular donor plasmids, and not linearized ones, work. This experimental strategy works for attached cells and EBV-transformed lymphoblastoid cell lines, which are suspension cells.

In summary, the experimental strategy is very straightforward. To clone EBV genomes in a specific EBV-positive cell line, one should co-transfect a pair of circular plasmids (pX330-sgEBV and a donor plasmid with appropriate homology arms), subject the transfected cells to hygromycin selection for three weeks, and wait for the appearance of GFP-positive cell colonies.

## 8. How to Pick Bacterial Colonies Harboring EBV-BAC Clones

Once homologous recombinants are detected in the episomal fractions, EBV-BAC clones can be readily obtained by transforming DH10B bacterial cells with episomal DNAs (Figure 1). The bacterial colonies obtained are screened by colony-direct-PCR for the presence of EBV IR1 sequences (BamHI W repeats). Under optimal experimental conditions, EBV-BACs can be obtained at a frequency of more than 40%, while other colonies will harbor the donor plasmid.

**Figure 4:**
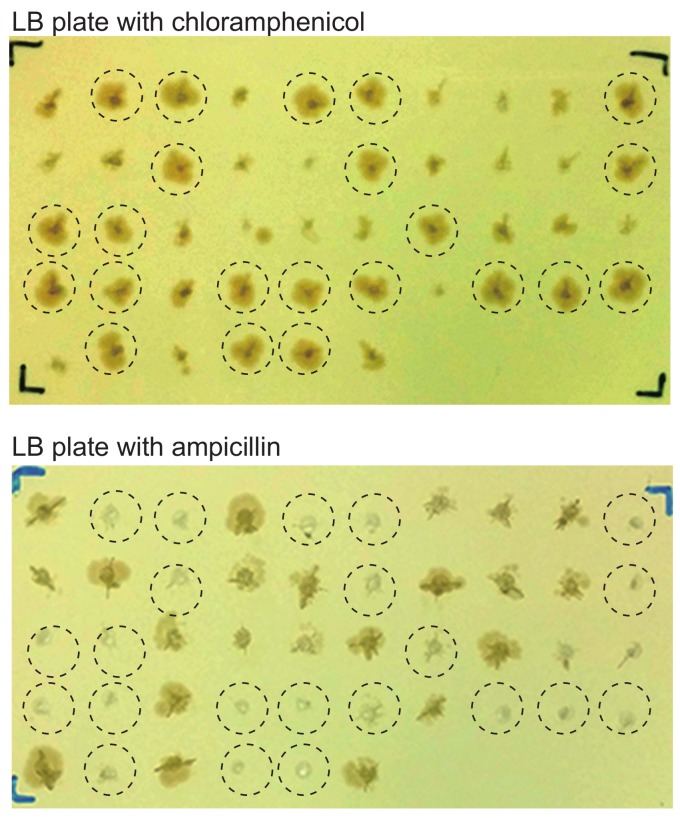
Bacterial colonies harboring EBV-BAC clones are larger than those harboring donor plasmids. EBV-positive gastric cancer cell line SNU-719 was subjected to EBV-BAC cloning. Individual chloramphenicol-resistant bacterial colonies obtained by transforming *E. coli* with episomal DNAs were picked up and transferred to LB plates containing either chloramphenicol (12.5 μg/mL) or ampicillin (200 μg/mL). Note that *E. coli* colonies growing faster on a chloramphenicol plate (**top**) are ampicillin-sensitive (**bottom**) (indicated by dotted circles).

When we examined the colonies of replica plates that were made for colony-direct PCR, we noticed that colonies harboring EBV-BACs were larger than those harboring donor plasmids (Figure 4). In other words, bacterial colonies harboring EBV-BAC clones appear to proliferate faster than those harboring donor plasmids. This is most likely due to the presence of dual replication origins (origin of pBluescript and origin of BAC vector) in the donor plasmid. The homologous recombinants retain only the origin from the BAC vector, because the Bluescript origin has been clipped out (Figure 2). Thus, after obtaining bacterial colonies derived from episomal DNAs, replica plates can be used to identify bacterial clones that are likely to be EBV-BAC clones. Furthermore, bacterial colonies harboring EBV-BAC clones are ampicillin-sensitive (Figure 4), as the clones lack the ampicillin-resistance gene. This is another way of selecting bacteria harboring EBV-BAC clones.

## 9. PacBio Sequencing of the BAC-Cloned EBV Genomes

The majority of whole EBV Genome sequencing studies have been done by subjecting genomic DNAs of EBV latently infected cells to short-read sequencers (Illumina HiSeq) [2]. However, short-read sequencers cannot determine the sequences of EBV repetitive regions [3,35]. Whole EBV genome sequence of M81 strain EBV was determined by subjecting EBV-BAC clone DNA to Illumina HiSeq 2000 (Illumina Inc., San Diego, CA, USA), followed by gap filling using a standard Sanger sequencing [10]. To circumvent the difficulty, we use PacBio single molecule real time (SMRT) sequencing technology, which is a long-read sequencer [36]. Although the accuracy of each read is approximately 85%, by getting read depth of more than 1000, one can obtain contigs with 99.999% accuracy. In our study, when we annotated SNU719-BAC and YCCEL1-BAC sequences, only one nucleotide mistake was found in each of the BAC clones [21]. Both mistakes were within the homonucleotide stretches. Thus, although PacBio sequencing produces very accurate results, special attention must be paid to homonucleotide stretches.

PacBio sequencing can determine complete EBV genome sequences including repetitive regions, such as IR1 and the FR sequences [21]. FR sequences are especially difficult to determine by short-read sequencers and Sanger sequencing. Thus, PacBio sequencing using EBV-BAC clone DNAs is so far the only method to readily obtain complete EBV genome sequences. Other long-read sequencers, such as minION (Oxford Nanopore Technologies, Oxford, UK) may also work. De novo assembly of herpesvirus type 1 genome was recently reported by using long reads (obtained by minION) as scaffold, and by using short reads (obtained by Roche 454) for sequence curation [37]. Whether minION can determine EBV repetitive sequences such as IR1 and FR should be clarified.

## 10. Reconstitution of Infectious Viruses

BAC cloning of various EBV strains enables reconstitution of infectious viruses and investigation of their phenotypes. Virus producing cells can be established by introducing EBV-BAC clone DNAs into HEK293 cells, followed by hygromycin selection. After picking hygromycin-resistant, GFP-positive cell colonies at 3–4 weeks post-transfection, the cells are grown and tested for their ability to produce progeny viruses. For some EBV-BACs so far tested, it is, for unknown reasons, very difficult to obtain virus-producing cells. We cannot completely exclude the possibility that unintended mutations are introduced while cloning the EBV genome into a BAC vector. The instability of FR sequence mentioned earlier is one of the possible reasons for the inconsistency of obtaining viral progenies. So far, it is easy to obtain viral progenies of B95.8-BAC, SNU719-BAC, and YCCEL1-BAC [7,21], all of which harbor intact FR sequences, while some EBV-BAC clones with truncated FR fail to produce their progenies. Further studies are necessary to clarify the reason for the inconsistency.

## 11. Problems to Be Solved in the Future

CRISPR/Cas9-mediated EBV-BAC cloning is a powerful tool that contributes greatly to EBV genetics. However, it will have to be further improved to allow its wider use in the future.

First, the efficiency of obtaining homologous recombinants should be further improved, especially in cells that are difficult to transfect. LCLs and EBV-positive NK/T lymphoma cell lines are difficult to transfect, thus the same CRISPR/Cas9 plasmid (pX330-sgEBV) may be inefficient. This problem could be potentially resolved by including the *oriP* sequence in the CRISPR/Cas9 plasmid. Including the *oriP* sequence would make the CRIPSR/Cas9 plasmid episomally maintained in EBV-infected cells, as viral protein EBNA1, a trans-acting *oriP* binding protein, is expressed in all the EBV-infected dividing cells [38]. The FR sequence of *oriP* also mediates nuclear retention of the transfected plasmids in EBNA1-dependent manner [39]. Indeed, including the *oriP* sequence in the CRISPR/Cas9 plasmid greatly improves its ability to cut the target sequence in EBV-positive cell lines [17]. Alternatively, rather than transducing CRISPR/Cas9 plasmids, delivery of purified Cas9 ribonucleoproteins could result in better efficiency [40]. Whether Cas9 RNPs efficiently enter LCLs or T-NK cells should be clarified. Efficient CRISPR/Cas9-mediated genome editing in LCLs was recently reported [41]. The study utilizes a lentiviral vector to stably express Cas9 protein in LCLs, followed by lentiviral transduction of single-guide RNA (sgRNA) library. Lentiviral transduction of Cas9 and sgRNA can also be applied to EBV-BAC cloning. Alternatively, the upstream homology arm of the donor plasmid (currently 230-bp) can be extended to ~800-bp, which may increase the efficiency of homologous recombination. The resultant homology arms are still much shorter than those used for the conventional targeting.

The second and more difficult issue is intrinsic to the EBV-BAC system. Some repetitive sequences within EBV genomes are unstable in *E. coli*; for example, the FR sequence is quite unstable in a BAC vector [7]. FR consists of multiple (20 to more than 30) copies of a 30-bp palindromic unit, each of which acts as a binding site for viral EBNA1 protein dimer [38]. The FR sequence can be maintained stably when the B95-8, SNU719, and YCCEL1 strains are cloned in a BAC vector [7,21]. However, for several lymphoblastoid cell lines, EBV-BACs with intact FR sequences could not be obtained. Since FR is a critical cis-acting sequence required for episomal maintenance of EBV episomes in latently infected cells [38], FR instability in the EBV-BAC clones may result in impaired episomal maintenance. Thus, it is critical to use EBV-BAC clones with intact FR sequences so that infectious viruses can be reconstituted for various functional assays. One possible solution is to restore FR sequences by using those that can be stably maintained in EBV-BAC clones, such as those of B95-8, SNU-19, and YCCEL1.

## 12. Applications of CRISPR/Cas9-Mediated Cloning of EBV

BAC cloning of EBV genomes, followed by whole viral genome sequencing by PacBio, enables further study of the global distribution of EBV strains. Naturally arising LCLs are available from various sources. For example, they can be established from peripheral blood [42] or from tonsillar tissues obtained by routine tonsillectomy [43]. These naturally arising LCLs can serve as ideal resources to investigate EBV strain variation among individuals. EBV genetic variation within asymptomatically infected individuals has never been investigated. Sequence variation among EBV strains mainly resides within viral latent genes [3]. We know that different EBV strains encode viral latent proteins with different sizes, and the size difference is due to the different copy numbers of repetitive amino acid motifs within them [21]. Furthermore, other repetitive sequences within EBV genomes, such as FR and other noncoding sequences, also exhibit sequence variation [44]. Complete viral genome sequences obtained by PacBio and other long-read sequencers would be extremely useful for the investigation of such sequence variation within repetitive regions.

We are also interested in examining clinical EBV isolates of other EBV-positive diseases, such as EBV-positive T/NK lymphoma cells and gastric cancer cells. EBV-positive T/NK lymphoma cell lines are available, and they can readily be subjected to BAC cloning using CRISPR/Cas9. Furthermore, based on recent technical progress in cultivating primary cancer cells [45], primary EBV-positive gastric cancer tissues can also be converted to cell lines and subjected to EBV-BAC cloning.

CRISPR/Cas9-mediated cloning of EBV genomes has opened up a new era in EBV genetics. Whether specific EBV strains are related to specific diseases can be investigated in the future [46]. Since disease-associated EBV strains can now be reconstituted as infectious viruses, the phenotypic differences between asymptomatically infecting EBV and disease-associated EBV can be examined. CRISPR/Cas9-mediated EBV cloning will accelerate the characterization of various EBV strains and allow us to test the hypothesis that EBV strain variation results in different pathogenicity.
